# Risk factors for Hodgkin's disease by Epstein-Barr virus (EBV) status: prior infection by EBV and other agents

**DOI:** 10.1054/bjoc.1999.1049

**Published:** 2000-02-01

**Authors:** F E Alexander, R F Jarrett, D Lawrence, A A Armstrong, J Freeland, D A Gokhale, E Kane, G M Taylor, D H Wright, R A Cartwright

**Affiliations:** Department of Public Health Science, University of Edinburgh, Medical School, Teviot Place, Edinburgh, EH8 9AG, UK; Department of Veterinary Pathology, Leukaemia Research Fund Virus Centre, Bearsden Road, Glasgow, G61 1QH, UK; Clinical Trial and Statistics Unit, Institute of Cancer Research, Block D, 15 Cotswold Road, Sutton, Surrey, SM2 5NG, UK; North West Immunogenetics Laboratory, St Mary's Hospital, Hathersage Road, Manchester, M13 0JH, UK; Leukaemia Research Fund, Centre for Clinical Epidemiology, UMV Leeds, 17 Springfield Mount, Leeds, LS2 9NG, UK; Department of Pathology, University of Southampton, Level E, South Block, Southampton General Hospital, Southampton, UK

**Keywords:** Hodgkin's disease, Epstein–Barr virus, aetiology, late host exposure model, infectious agents

## Abstract

A UK population-based case–control study of Hodgkin's disease (HD) in young adults (16–24 years) included 118 cases and 237 controls matched on year of birth, gender and county of residence. The majority (103) of the cases were classified by Epstein–Barr virus (EBV) status (EBV present in Reed–Stenberg cells), with 19 being EBV-positive. Analyses using conditional logistic regression are presented of subject reports of prior infectious disease (infectious mononucleosis (IM), chicken pox, measles, mumps, pertussis and rubella). In these analyses HD cases are compared with matched controls, EBV-positive cases and EBV-negative cases are compared separately with their controls and formal tests of differences of association by EBV status are applied. A prior history of IM was positively associated with HD (odds ratio (OR) = 2.43, 95% confidence interval (CI) = 1.10–5.33) and with EBV-positive HD (OR = 9.16, 95% CI = 1.07–78.31) and the difference between EBV-positive and EBV-negative HD was statistically significant (*P* = 0.013). The remaining infectious illnesses (combined) were negatively associated with HD, EBV-positive HD and EBV-negative HD (in the total series, for ≥2 episodes compared with ≤1, OR = 0.45, 95% CI = 0.25–0.83). These results support previous evidence that early exposure to infection protects against HD and that IM increases subsequent risk; the comparisons of EBV-positive and EBV-negative HD are new and generate hypotheses for further study. © 2000 Cancer ResearchCampaign


British Journal of Cancer (2000) 82(5), 1117–1121
© 2000 Cancer Research Campaign
DOI: 10.1054/ bjoc.1999.1049, available online at http://www.idealibrary.com on IDEAL®

### Risk factors for Hodgkin's disease by Epstein-Barr virus (EBV) status: prior infection by EBV and other agents

**FE Alexander[1], RF Jarrett[2], D Lawrence[3], AA Armstrong[2], J Freeland[2], DA Gokhale[4], E Kane[5], GM Taylor[4], DH Wright[6] and RA Cartwright[5]**

[1]Department of Public Health Science, University of Edinburgh, Medical School, Teviot Place, Edinburgh EH8 9AG, UK; [2]Leukaemia Research Fund Virus Centre, Department of Veterinary Pathology, Bearsden Road, Glasgow G61 1QH, UK; [3]Clinical Trial and Statistics Unit, Institute of Cancer Research, Block D, 15 Cotswold Road, Sutton, Surrey SM2 5NG, UK; [4]North West Immunogenetics Laboratory, St Mary's Hospital, Hathersage Road, Manchester M13 0JH, UK; [5]Leukaemia Research Fund, Centre for Clinical Epidemiology, UMV Leeds, 17 Springfield Mount, Leeds LS2 9NG, UK; [6]Department of Pathology, University of Southampton, Level E, South Block, Southampton General Hospital, Southampton, UK

**Summary** A UK population-based case–control study of Hodgkin's disease (HD) in young adults (16–24 years) included 118 cases and 237 controls matched on year of birth, gender and county of residence. The majority (103) of the cases were classified by Epstein–Barr virus (EBV) status (EBV present in Reed–Stenberg cells), with 19 being EBV-positive. Analyses using conditional logistic regression are presented of subject reports of prior infectious disease (infectious mononucleosis (IM), chicken pox, measles, mumps, pertussis and rubella). In these analyses HD cases are compared with matched controls, EBV-positive cases and EBV-negative cases are compared separately with their controls and formal tests of differences of association by EBV status are applied. A prior history of IM was positively associated with HD (odds ratio (OR) = 2.43, 95% confidence interval (CI) = 1.10–5.33) and with EBV-positive HD (OR = 9.16, 95% CI = 1.07–78.31) and the difference between EBV-positive and EBV-negative HD was statistically significant (*P* = 0.013). The remaining infectious illnesses (combined) were negatively associated with HD, EBV-positive HD and EBV-negative HD (in the total series, for ≥2 episodes compared with ≤1, OR = 0.45, 95% CI = 0.25–0.83). These results support previous evidence that early exposure to infection protects against HD and that IM increases subsequent risk; the comparisons of EBV-positive and EBV-negative HD are new and generate hypotheses for further study. © 2000 Cancer Research Campaign

**Keywords**: Hodgkin's disease; Epstein–Barr virus; aetiology; late host exposure model; infectious agents

The age-incidence curve for Hodgkin's disease (HD) in developed countries includes a striking peak amongst young adults (aged 16–34 years) which, in 1966, led to the hypothesis that there are three distinct HD entities corresponding to childhood, young adult and older age-at-onset (MacMahon, 1966) with an 'infectious aetiology' for young adult HD. Considerable support has amassed for the 'late host response model' (Gutensohn and Cole, 1980) of HD in young adults under which the disease is a (rare) sequella to late first infection by one or more unknown infectious agents. The evidence includes ecological, case–control and cohort studies showing positive associations between HD in young adults and proxies for risk of late exposure to infections or absence of early exposure to 'marker' agents (reviewed in Mueller and Grufferman, 1999). Further support has come from reports (Miller and Beebe, 1973; Connelly and Christine, 1974; Rosdahl et al, 1974; Carter et al, 1977; Munoz et al, 1978; Kvale et al, 1979) that incidence of HD is high in cohorts of subjects who have had infectious mononucleosis (IM); these studies involve nearly 42 000 young adults with serologically confirmed IM and, overall, show risk elevated around threefold. IM is caused by late first infection with the Epstein–Barr virus (EBV) and its age-incidence curve parallels that of HD in young adults.

*Received 6 May 1999*
*Revised 18 October 1999*
*Accepted 19 October 1999*

*Correspondence to:* FE Alexander

The demonstration that EBV genomes were present and expressed in the HD tumour cells (Reed–Sternberg cells) of a proportion of cases provided an important new understanding of the biology of the disease (Weiss et al, 1987; Pallesen et al, 1991). It is now known that EBV is present in around 40% of all cases of HD (Jarrett et al, 1996) and its role is generally agreed to be causal although details of the relationship remain poorly understood (Michels, 1995; Mueller, 1996). Subclassification of cases of HD by presence (EBV-positive) or absence (EBV-negative) of EBV in Reed–Stenberg cells provides a biological classification which is alternative to the histological grouping by Rye type and has the potential to identify aetiological subgroups but only one study (a case-series) has compared epidemiological risk factors by EBV status (Sleckman et al, 1998). The objective of the present study was to investigate the late host response model for HD in young adults (16–24 years) and determine whether it applied differently to EBV-positive and EBV-negative disease. The present report focuses on self-reported history of IM and other specific infectious illness. A subsequent paper will report HLA-DP type of cases by EBV status.

### METHODS

### Cases

All cases of HD newly diagnosed between 1 October 1991 and 31 May 1995 in the Yorkshire, Wessex and South West FHSA areas and in Cumbria and Lancashire (part), and aged 16–24 years at diagnosis were eligible for inclusion. These areas are taken from the Leukaemia Research Fund (LRF) Data Collection Study (DCS) area and methods of ascertainment were those of the DCS (Cartwright et al, 1990). This ensures high quality ascertainment free of geographical bias. Altogether 129 cases were eligible and 118 consented to participate.

### Controls

Controls (two per case) were randomly selected from people registered with general practitioners in the study area matched for sex, year of birth and administrative area (FHSA) of residence. This random selection from computerized general practitioner (GP) lists will have included an unknown number of people who were no longer living at the registered address (or even in the FHSA); such people were not eligible for the study. Controls were approached by letter after their GPs had given consent; second letters were sent to those who did not respond and further attempts to make contact included telephone calls and home visits. Controls who did not give consent or who could not be traced were replaced with further random selection based on the same matching criteria and using the same methods of approach.

### Socio-economic status

The address of residence has been used to give the Carstair's index (Jarman et al, 1991) of area of residence as an indicator at five levels of socio-economic status using data derived from the 1991 census.

### Interview data

Face-to-face interviews were conducted by trained interviewers using questionnaires developed from others in use by the LRF Centre for Clinical Epidemiology in Leeds (Cartwright et al, 1990). The period covered was from birth up to 'reference date', which was date of diagnosis of the case for members of each matched set. Information was elicited on proxies for exposure to infection (not reported here), past history of infectious illness, past medical history of index and family, and limited history of infectious illness in friends and household members. Each subject was asked 'Have you ever suffered from glandular fever?' and the response was recorded as 'Yes', 'No', 'It was suspected' or 'Not known'. Self-reported IM (glandular fever) has been analysed in two ways taking 'suspected' as 'Yes' and as 'No'; taking 'suspected' as missing led to too much exclusion of data under the matched design. Since symptoms of IM can arise as a preliminary indication of HD, analyses for both total IM before reference date and IM up to 1 year before reference date are reported.

Reported history of 'childhood infectious illness' (measles, rubella, mumps, chicken pox and pertussis) was available for almost all subjects. A blind assessment of the distribution of frequencies of childhood infections led to sensible strata for the number of episodes for analyses. The analyses reported here in detail are all based on dichotomies in which the lowest level (usually none) is taken as reference group and all others combined. A decision was taken prior to inspection of the data that childhood infections should be considered at all ages and in 5-year age groups. Since few infectious illnesses occurred in children over 10 years, some analyses have taken ≥ 5 as a single age group.

### EBV status classification and histopathology

Paraffin-embedded biopsy material was retrieved from cases and histopathological review was performed by DHW. Sections were examined for the presence of EBV using EBV EBER in situ hybridization and also, in the majority of cases, LMP-1 immunohistochemistry. The in situ hybridization assay utilized a biotinylated oligonucleotide probe complementary to the EBER-1 RNA which has been described previously (Armstrong et al, 1992). Hybridization was detected using avidin–biotin complexes, and nitroblue tetrazolium was used as the chromogenic substrate (Dako, High Wycombe, UK). Expression of the LMP-1 protein was investigated using the CS1–4 cocktail of monoclonal antibodies as previously described (Armstrong et al, 1992). Sections from known cases of EBV-positive associated HD were used as positive controls in both assays. Cases are described as EBV-positive if the Reed–Sternberg cells scored positive in either assay. EBV status is available for 103 and histological review for 105 of the cases, including all but one of those with EBV status known. All but four cases have been given a specific Rye type but those with type given as lymphocyte predominant (LP) have been excluded from some analyses because they are not now considered 'classical' HD.

### Statistical analysis

Almost all statistical analyses have applied conditional logistic regression to the matched set data to compare risk factors for cases and controls with results reported as odds ratios (OR) and 95% confidence intervals (CI). These have been implemented in the software packages SAS and EGRET. Multivariate conditional logistic regression has permitted adjustment for confounding variables and for Carstair's index of address of residence. Analyses have been applied to the following subgroups:

- all subjects
- all sets where case EBV status is known, with testing for interaction by EBV status of the case.

Where the conditional logistic model could not be fitted (0 subjects in one cell) exact analyses (with matching retained) have been conducted in EGRET.

All testing of statistical significance for conditional logistic regression modelling has examined the deviance difference against its asymptotic chi-square distribution under the null hypothesis (Clayton and Hills, 1993). Main effects were mostly tested against a $\chi^2$ distribution with one degree of freedom; for heterogeneity of risk by case EBV-status subgroups the $\chi^2$ for the interaction had a further 1 degree of freedom. The hierarchy of hypotheses considered here is: no association, association common to both case subgroups, association different by case subgroup.

### RESULTS

The majority (90%) of cases ascertained in the geographic area during the study period were recruited into the study. Cases (Table 1) were predominantly of NS type and predominantly EBV-negative. Overall, there was a very slight excess of males but the EBV-positive cases showed a substantial male excess (14 males, five females).

© 2000 Cancer Research Campaign

**Table 1** Numbers of cases for analysis with selected characteristics

| **Characteristics** | | ***N* (%)** |
|---|---|---|
| Gender | Male | 62 (52.5) |
| | Female | 56 (47.5) |
| Rye type | NS | 81 (68.6) |
| | MC | 14 (11.9) |
| | LD | 1 (0.8) |
| | LP | 14 (11.9) |
| | NOS | 4 (3.4) |
| EBV status | Positive | 19 (16.1) |
| | Negative | 84 (71.2) |

[a] % of all cases; where data are missing %s do not add up to 100.
NS, Nodular sclerosing
MC, Mixed cellularity
LD, Lymphocyte depleted
LP, Lymphocyte predominant
NOS Not otherwise specified

**Table 2** Association of reported IM with HD case status

| **Definition of 'exposed'** | **Total series** % Positive[a] Ca | Ct | OR[b] (95% CI) | *P* | **EBV-positive cases** % Positive[a] Ca | Ct | OR[b] (95% CI) | *P* | **EBV-negative cases** % Positive[a] Ca | Ct | OR[2] (95% CI) | *P* |
|---|---|---|---|---|---|---|---|---|---|---|---|---|
| Definite prior IM | 16.1 | 9.0 | 2.43 (1.10–5.33) | 0.027 | 31.6 | 8.1 | 9.16[+] (1.07–78.31) | 0.043 | 13.1 | 9.0 | 1.60 (0.63–4.07) | 0.32 |
| Definite or suspected prior IM | 21.2 | 13.7 | 1.87 (0.95–3.66) | 0.07 | 31.6 | 8.1 | 9.16[+] (1.07–78.31) | 0.043 | 19.0 | 13.3 | 1.50 (0.68–3.33) | 0.32 |
| Definite IM > 1 year before diagnosis | 13.6 | 8.2 | 1.93 (0.87–4.28) | 0.11 | 31.6 | 5.4 | ∞[c] (1.66–∞) | 0.011[c] | 9.5 | 8.4 | 1.09 (0.40–2.97) | 0.87 |
| Definite or suspected IM > 1 year before diagnosis | 17.8 | 12.9 | 1.46 (0.72–2.94) | 0.29 | 31.6 | 5.4 | ∞[c] (1.66–∞) | 0.011[c] | 14.3 | 12.7 | 1.00 (0.42–2.41) | 1.00 |

[a] % reporting positive of those with results available for the present analysis. [b]Adjusted for Carstairs index (except where this leaves 0 cases or 0 controls exposed for one exposure category (+) where unadjusted results are reported). [c]Exact tests performed maintaining the matching.

Recruitment of controls proved difficult in this age group. The percentage of first choice controls recruited was 36.5%; 39% of females but just 31% of males who were approached consented to participate. Approximately half of the non-participants were eligible but refused consent. We had no way of verifying the eligibility of the remainder. Our recorded response rates may therefore be artefactually low. Control response was associated with Carstair's index with 45% of those approached in the two least deprived groups consenting compared with 25% in the most deprived group. Subsequent analyses therefore have been adjusted for Carstair's index whenever possible; since the index could not be calculated from the recorded addresses of 20 cases and 38 controls this has led to the exclusion of a small amount of data.

When frequencies of reported IM in cases and controls were compared (Table 2) there was a significant case excess in the total series when 'suspected' was interpreted as 'no'. The magnitude of the OR and the level of statistical significance was reduced if 'suspected' was taken as 'yes' and/or IM in the last year before reference date was ignored. The OR are much higher when the EBV-positive cases are compared with their controls and no comparisons achieve statistical significance when analyses are restricted to the EBV-negative cases. Statistical testing of the interaction confirms heterogeneity of the OR by case EBV-status ($P$ = 0.013 for definite and 0.009 for definite or suspected IM prior to diagnosis).

Childhood infectious illnesses were significantly protective for the total case series and for EBV-positive HD and EBV-negative HD analysed separately (Table 3). Infections at ages 5–9 years were significantly protective for EBV-negative HD but appeared to increase risk of EBV-positive HD and the interaction with case EBV status achieved formal statistical significance ($P$ = 0.02). Measles (but not any other of the individual illnesses) was significantly protective for the total series.

Further examination of combined infections at ages 5–9 years was appropriate because of the significant interaction with EBV status. Scrutiny of the data revealed that the (non-significant) excess risk of EBV-positive HD associated with infections at ages 5–9 years was focused in the IM-positive subjects. The interaction of IM with combined infection at this age was tested in the total series and found to be statistically significant (Table 4). Thus, the data suggest that people with IM who have also had a school-age history of other infectious illness are at special risk of HD (particularly EBV-positive). Numbers of EBV-positive cases were too small to permit these analyses for EBV-positive cases alone; we did, however, confirm that the effect of IM in the EBV-positive subjects persisted after adjustment for infections at ages 5–9 years (minor reduction in OR in both the conditional logistic model and exact analyses).

The five childhood infectious illnesses were considered individually in two age groups (younger: < 5 years, older: ≥ 5 years). When all these were included in a multivariate analysis of the total series older measles was significantly protective (OR = 0.32; 95% CI = 0.14–0.74, adjusting for younger measles and the other infections in two age groups). No other comparisons approached statistical significance. When a similar analysis was conducted for the EBV-negative cases (and their controls) the results for measles were similar but older chicken pox was now protective to a similar extent (OR = 0.39, 95% CI 0.17–0.93). The numbers of EBV-positive cases were too small for the multivariate analysis to be applied. Addition of IM to the two full models (i.e. those containing terms for five infections each at two ages) made some improvement for the total series (OR = 1.86, 95% CI = 0.71–4.84, $P$ = 0.20) but had a very small effect for the EBV-negative series (OR = 1.18, 95% CI = 0.39–3.52, $P$ = 0.77) so that IM has virtually no independent effect on risk of EBV-negative HD.

*© 2000 Cancer Research Campaign*

**Table 3** Association of selected reported infections with HD case status

| Definition of 'exposed' | Total series | | | | EBV-positive cases | | | | EBV-negative cases | | | |
|---|---|---|---|---|---|---|---|---|---|---|---|---|
| | % positive[a] Ca | % positive[a] Ct | OR[b] (95% CI) | *P* | % positive[a] Ca | % positive[a] Ct | OR[b] (95% CI) | *P* | % positive[a] Ca | % positive[a] Ct | OR[2] (95% CI) | *P* |
| Total *n* infections ≥ 2 (see Table 2) | 68.6 | 81.8 | 0.45 (0.25–0.83) | 0.010 | 63.2 | 86.5 | 0.18 (0.03–0.95) | 0.043 | 66.7 | 81.7 | 0.43 (0.21–0.86) | 0.017 |
| Total *n* infections < 5 years ≥ 1 | 37.3 | 40.7 | 0.89 (0.54–1.49) | 0.66 | 26.3 | 43.2 | 0.32 (0.07–1.49) | 0.15 | 40.5 | 39.6 | 1.01 (0.55–1.88) | 0.97 |
| Total *n* infections 5–9 years ≥ 1 | 72.9 | 78.5 | 0.82 (0.47–1.42) | 0.48 | 84.2 | 70.3 | 4.70 (0.55–40.22) | 0.16 | 66.7 | 81.1 | 0.51 (0.27–0.98) | 0.042 |
| Total *n* infections ≥ 10 years ≥ 1 | 16.9 | 20.3 | 0.85 (0.44–1.63) | 0.62 | 31.6 | 21.6 | 2.31 (0.57–9.37) | 0.24 | 15.5 | 21.9 | 0.62 (0.27–1.39) | 0.24 |
| Measles ever | 37.8 | 53.3 | 0.53 (0.32–0.90) | 0.018 | 47.4 | 58.3 | 0.48 (0.13–1.81) | 0.28 | 34.6 | 51.9 | 0.49 (0.27–0.90) | 0.020 |

[a] % reporting positive of those with results available for the present analysis. [b]Adjusted for Carstairs index (except where this leaves 0 cases or 0 controls exposed for one exposure category (+) where unadjusted results are reported).

**Table 4** Interaction of reported IM and total infections at ages 5–9 years (using total series)

| Exposure (term in model) | OR | 95% CI |
|---|---|---|
| Reported IM (suspected = No) | 0.47 | 0.09–2.52 |
| Total *n* infections (as in Table 2) ≥1 ages 5–9 years | 0.64 | 0.37–1.10 |
| Interaction (reported IM and ≥ 1 infection 5–9 years) | 5.84 | 0.99–34.59 |

*P*-value for statistical interaction: 0.035.

### DISCUSSION

The study possesses unique strengths; these include the cases being a population-based census of all those arising in a narrow age range and the systematic ascertainment of Rye-type and EBV-status of cases. No previous epidemiological case–control study of HD has included all of these classifications. The results present the first evidence of an association between prior IM and EBV-positive HD.

Our study, however, has two important limitations. First, it is based on small numbers, especially of EBV-positive cases. Secondly, a large number of first-choice controls could not be recruited; this may have led to bias between participating controls and controls selected so that the former are not representative of the population from which the cases derive. We have evidence that, in particular, controls recruited are of higher socio-economic status. Whilst acknowledging these limitations we emphasize that, in regard to the first point, this study provides preliminary conclusions for subsequent testing. With regard to control recruitment we note that adjustment for Carstair's index will reduce the problem but, critically, the bias cannot affect comparisons by biological subgroups of cases. A further potential weakness is absence of medical case-note or serological verification of IM. A recent large case–control of several cancers including HD has found ORs for self-reported IM which were similar to those from the cohort studies, and show disease specificity and time-period specificity which were consistent with the literature (Levine et al, 1998). This suggests that recall bias is of limited importance. Also, it is clear that any such effects cannot apply to comparisons of the EBV subgroups of HD.

There are three separate strands of evidence relating EBV to HD: past history of IM in HD cases; serological studies comparing EBV antibody titres in HD cases and controls; molecular biological studies which have demonstrated presence and expression of EBV in HD tumour cells for around 40% of cases (Jarrett et al, 1996). The above three strands have not been critically compared with each other.

Previous IM is associated with young adult HD, whereas EBV positivity is rare in this age group (Glaser et al, 1997). The evidence associating prior IM with HD is open to two interpretations which are not mutually exclusive: EBV infection may have a causal role in the subsequent HD *or* late first infection by EBV (resulting in IM) may indicate a lifestyle in which first infection by a broad range of agents is delayed to young adulthood. Limited data have been published for EBV status of HD cases with prior IM (Mack et al, 1995; Sleckman et al, 1998) but EBV-negative cases certainly occur in people with a history of IM. The first study to have compared risk factors in EBV-positive and -negative HD cases was a case-series (Sleckman et al, 1998) which found no association between prior IM and EBV status; this study had similar numbers of cases to our own but included a much broader range of ages at diagnosis (16–55 years).

Our most important results are evidence that reported IM (i) is statistically significantly associated with HD, (ii) is focused in EBV-positive cases, and (iii) has a statistically significant interaction with EBV status of cases. Our results suggest a specific causal association of recent EBV exposure with EBV-positive HD which may be superimposed on an additional risk related to a lifestyle conducive to late first exposure to infection.

Our data are consistent with *either* an absence of association of EBV-negative HD with prior IM *or* a weak positive association; the latter could be interpreted in terms of lifestyles and environments predisposing to late first exposures to EBV and other agents with similar transmission routes. It is also important to note that our data document for the first time the high frequency of reported IM in young adults in the UK (and similar countries) today. This could in itself explain the anecdotal reports of prior IM in EBV-negative HD.

Evidence for a protective role for (early exposure to) non-specific infectious agents in the aetiology of HD comes largely from proxy data (see Introduction), although one cohort study with baseline data being a detailed history of prior infectious illness reported by 1st year university students, reached similar conclusions (Paffenbarger et al, 1977). Measles and/or combined child-

*© 2000 Cancer Research Campaign*

hood infections are protective for HD in our data; biological considerations and the HD epidemiological profile suggest that this is due to their being markers of reduced risk of late first exposure to aetiological agents. The present data cannot distinguish between measles and total infections but are consistent with a specific protective effect of measles in school age children.

Although, in general, infection is protective against both EBV-positive and EBV-negative HD there is evidence that the relevance of childhood infectious illnesses at specific ages differs by EBV status. Total infectious illness in children aged 5–9 years has a negative association with EBV-negative HD but a positive one with EBV-positive disease (these results being driven by chicken pox). The elevated OR and the statistically significant interaction between EBV-positive HD and EBV-negative HD may be (i) due to chance or (ii) indicative of a genuine association, possibly involving an underlying synergism with IM. The latter is supported by the statistically significant interaction in which risk of HD is concentrated in individuals who reported both IM and childhood infectious illness (5–9 years).

The present study generates hypotheses for testing in larger series. Several of us are collaborating in a much larger study of HD in subjects 16–74 years. The same risk factor and biological data are available in this larger study and analyses will begin shortly. The results point to the importance of recent exposure to EBV in the development of EBV-positive HD in young adults.

### REFERENCES

Armstrong AA, Weiss LM, Gallagher A, Jones DB, Krajewski AS, Angus B, Brown G, Jack AS, Wilkins BS and Onions DE (1992) Criteria for the definition of Epstein–Barr virus association in Hodgkin's disease. *Leukemia* 6: 869–874

Carter CD, Brown TM Jn, Herbert JT and Heath CW (1977) Cancer incidence following infectious mononucleosis. *Am J Epidemiol* **105**: 30

Cartwright RA, Alexander FE, McKinney PA, Ricketts TJ, Hayhoe FGJ and Clayton DGC (1990) *Leukaemia and Lymphoma: An Atlas of Distribution Within Areas of England and Wales, 1984–88*. Leukaemia Research Fund: London

Clayton D and Hills M (eds) (1993) *Statistical Methods in Epidemiology*. Oxford University Press: New York

Connelly RR and Christine BW (1974) A cohort study of cancer following infectious mononucleosis. *Cancer Res* **34**: 1172

Glaser SL, Lin RJ, Stewart SL, Ambinder RF, Jarrett RF, Brousset P, Pallesen G, Gulley ML, Khan G, OGrady J, Hummel M, Preciado MV, Knecht H, Chan JKC and Claviez A (1997) Epstein–Barr virus-associated Hodgkin's disease: epidemiologic characteristics in international data. *Int J Cancer* **70**: 375–382

Gutensohn N and Cole P (1980) Epidemiology of Hodgkin's disease. *Semin Oncol* **7**: 92

Jarman B, Townsend P and Carstairs V (1991) Deprivation indices. *Br J Med* **303**: 523

Jarrett RF, Armstrong AA and Alexander FE (1996) Epidemiology of EBV and Hodgkin's lymphoma. *Ann Oncol* **7**: S5–S10

Kvale G, Hoiby EA and Pedersen E (1979) Hodgkin's disease in patients with previous infectious mononucleosis. *Int J Cancer* **23**: 593

Levine R, Zhu KM, Gu Y, Brann E, Hall I, Caplan L and Baum M (1998) Self-reported infectious mononucleosis and 6 cancers. A population based case–control study. *Scand J Inf Dis* **30**: 211–214

Mack TM, Cozen W, Shibata DK, Weiss LM, Nathwani BN, Hernandez AM, Taylor CR, Hamilton AS, Deapen DM and Rappaport EB (1995) Concordance for Hodgkin's disease in identical twins suggesting genetic susceptibility to the young adult form of the disease. *N Engl J Med* **332**: 413–418

MacMahon M (1966) Epidemiology of Hodgkin's disease. *Cancer Res* 26: 1189

Miller RW and Beebe GW (1973) Infectious mononucleosis and the empirical risk of cancer. *J Natl Cancer Inst* **50**: 315

Michels KB (1995) The origins of Hodgkin's disease. *Eur J Cancer Prev* **4**: 379–388

Mueller NE (1996) Hodgkin's disease. In: *Cancer Epidemiology and Prevention*, 2nd edn, Schottenfield D and Fraumeni JF Jr (eds). Oxford University Press: New York

Mueller NE and Grufferman S (1999) Epidemiology. In: *Hodgkin's Disease*, Mauch P, Armitage J, Diehl V, Hoppe R and Weiss L (eds) Raven Press: New York (in press)

Munoz N, Davidson RJL, Witthoff B, Ericsson JE and De-The G (1978) Infectious mononucleosis and Hodgkin's disease. *Int J Cancer* **22**: 10

Paffenbarger RS Jn, Wing AL and Hyde RT (1977) Characteristics in youth indicative of adult-onset Hodgkin's disease. *J Natl Cancer Inst* **58**: 1489

Pallesen G, Hamilton-Dutoit SJ, Rowe M and Young LS (1991) Expression of Epstein–Barr virus latent gene products in tumour cells of Hodgkin's disease. *Lancet* **337**: 320–322

Rosdahl N, Larsen SO and Clemmenson J (1974) Hodgkin's disease in patients with infectious mononucleosis: 30 years experience. *Br Med J* **2**: 253

Sleckman BG, Mauch PM, Ambinder RF, Mann R, Pinkus GS, Kadin ME, Sherburne B, PerezAtayde A, Thior I and Mueller N (1998) Correlation between EBV-status and risk factor profile in Hodgkin's disease. *Cancer Epidemiol Biomark Prev* **7**: 1117–1121

Weiss LM, Strickler JG, Warnke RA, Purtilo DT and Sklar J (1987) Epstein–Barr viral DNA in tissues of Hodgkin's disease. *Am J Pathol* **129**: 86–91

© 2000 Cancer Research Campaign
